# Evaluation of Biological Pretreatment of Rubberwood with White Rot Fungi for Enzymatic Hydrolysis

**DOI:** 10.3390/ma6052059

**Published:** 2013-05-15

**Authors:** Forough Nazarpour, Dzulkefly Kuang Abdullah, Norhafizah Abdullah, Reza Zamiri

**Affiliations:** 1 Institute of Bioscience; University Putra Malaysia (UPM), 43400 Serdang Selangor, Malaysia; E-Mails: forough.nazarpour84@gmail.com (F.N.); fizah@eng.upm.edu.my (N.A.); 2 Department of Chemistry, Faculty of Science, University Putra Malaysia (UPM), 43400 Serdang Selangor, Malaysia; 3 Department of Chemical and Engineering, Faculty of Engineering, University Putra Malaysia (UPM), 43400 Serdang Selangor, Malaysia; 4 Department of Materials Engineering and Ceramic, University of Aveiro, Campus Santiago, 3810-193 Aveiro, Portugal; E-Mail: rezaz@ua.pt

**Keywords:** rubberwood, white rot fungi, biological pretreatment, enzymatic hydrolysis, XRD, FT-IR

## Abstract

The effects of biological pretreatment on the rubberwood (*Hevea brasiliensis*), was evaluated after cultivation of white rot fungi *Ceriporiopsis subvermispora*, *Trametes versicolor*, and a mixed culture of *C. subvermispora* and *T. versicolor*. The analysis of chemical compositions indicated that *C. subvermispora* had greater selectivity for lignin degradation with the highest lignin and hemicellulose loss at 45.06% and 42.08%, respectively, and lowest cellulose loss (9.50%) after 90 days among the tested samples. X-ray analysis showed that pretreated samples had a higher crystallinity than untreated samples. The sample pretreated by *C. subvermispora* presented the highest crystallinity of all the samples which might be caused by the selective degradation of amorphous components. Fourier transform infrared (FT-IR) spectroscopy demonstrated that the content of lignin and hemicellulose decreased during the biological pretreatment process. A study on hydrolysis of rubberwood treated with *C. subvermispora*, *T. versicolor*, and mixed culture for 90 days resulted in an increased sugar yield of about 27.67%, 16.23%, and 14.20%, respectively, as compared with untreated rubberwood (2.88%). The results obtained demonstrate that rubberwood is a potential raw material for industrial applications and white rot fungus *C. subevermispora* provides an effective method for improving the enzymatic hydrolysis of rubberwood.

## 1. Introduction

Rubberwood is one of the most abundant lignocellulosic materials in Malaysia. Rubber tree (*Hevea brasiliensis*), which is also known as *hevea* wood, is a major industrial crop grown in Southeast Asia with an estimated plantation area of 1.8 million ha (20% of global plantation) in Malaysia alone [1]. Rubberwood can be used as a potential raw material for bioethanol production due to its high cellulose content [2].

Enzymatic hydrolysis is an important step in conversion of inexpensive lignocellulosic materials to ethanol production. However, the recalcitrant nature of the lignocelluloses limits the access of hydrolytic enzymes to cellulose and hemicellulose components [3,4]. Existing pretreatment methods have largely been developed on the basis of physicochemical technologies such as steam explosion, dilute acid, alkali, and oxidation or varied combinations [5] but these processes usually require high temperature and operating pressure [6]. In contrast, microbial pretreatment utilizes microorganisms and their enzyme systems to degrade lignin and hemicellulose present in the lignocellulosic biomass with comparatively low energy and mild environmental conditions [3]. This environmentally friendly approach has recently received increasing attention [7].

White rot fungi are the only microorganisms that are able to efficiently degrade all the components of plant cell walls, both carbohydrates and lignin. Several species, e.g., *Ceriporiopsis subvermispora* and *Trametes versicolor* have been studied in great detail as model organisms for this complex process. The lignin-degrading system of these fungi is composed of extracellular enzymes together with low-molecular-mass cofactors [8,9]. Typically found ligninolytic enzymes are lignin peroxidase (LiP), manganese peroxidase (MnP), and laccase. *C. subvermispora* produces several MnP and laccase isoforms, but no lignin peroxidase. *T. versicolor* is the only one of these model organisms known so far to express all three of these ligninolytic enzymes efficiently [10]. Together with the cellulolytic enzyme system, these patterns of enzyme activities cause varied degrees of lignin and cellulose breakdown at different cultivation stages. The simultaneous attack of cellulose and lignin is the preferred strategy of *T. versicolor*, whereas *C. subvermispora* is a selective delignifier in the first stages of biotreatment, secreting only low activities of cellulolytic enzymes at a late culture stage [11,12], and apparently lacks cellobiohydrolase activity [12]. However, the effects on lignin-degrading abilities of selectively lignin-degrading white rot fungi in mixed cultures have received little attention [13,14]. Many of these mixed cultures were reportedly more efficient in lignocellulolytic biodegradation in producing high activity enzymes due to their synergistic action [14,15]. Mixed fungal cultures could lead to a higher enzyme production through synergistic interactions, but the final result seems to depend on the particular species combination or on the mode of interaction between species, and on the microenvironmental or nutritional conditions in the substrate under colonization [16]. Hence, the main goal of the present work was to investigate the potential of two white rot fungi, both individually and in a mixed culture, for the enhancement of enzymatic hydrolysis during 90 days of biological pretreatment. To date, no such work has been reported. Subsequently, the biodegradation patterns of the pretreated woods were also evaluated by wood component losses, X-ray diffraction, and Fourier Transform Infrared (FT-IR) analysis.

## 2. Experimental Section

### 2.1. Microorganisms and Inoculation

The fungi *Ceriporiopsis subvermispora* (ATCC 90467) and *Trametes (Coriolus) versicolor* (ATCC 20869) were purchased from American Type Culture Collection (ATCC) and maintained as a frozen culture (–80 °C) in 30% glycerol. The strains were pre-cultured on 2.4% potato-dextrose agar (PDA) plates at 28 °C for 7 days.

### 2.2. Biomass Preparation

The wood was chipped using the lab scale chipper. The chips then were transferred to a Pallman disc flaker and cut to smaller particle size. After flaking, they were ground to pass through a 1 mm screen and approximately dried to 5% moisture content in an oven at 103 ± 2 °C for 24 hours.

### 2.3. Biological Pretreatment with White Rot Fungi

Pretreatments were carried out in 250 mL Erlenmeyer flasks. Seven grams of rubberwood with 1 mm particle size were supplemented with 12 mL distilled water to obtain the appropriate substrate moisture content (75%). The flasks were sterilized at 121 ºC for 20 min then cooled and aseptically inoculated with a plug from the plate culture to obtain the effect of each fungus, individually and in a mixed culture, on the rubberwood. Parafilm was wrapped around flasks to act as a barrier against moisture loss and contamination. Small perforations were made to the film to avoid moisture condensation and allow ventilation of chambers. Flasks were maintained statically at 28 °C for 30, 60 and 90 days. A set of non-pretreated sterilized woods were used as control. After pretreatment, the flasks were stored at 4 °C before composition analysis and enzymatic hydrolysis. All experiments were carried out at least in triplicate.

### 2.4. Enzyme Hydrolysis

Enzymatic hydrolysis was performed following NREL laboratory analytical procedure LAP-008 [17]. Enzymatic hydrolysis experiments were carried out using commercial cellulase (Celluclast 1.5 L, produced by *Trichoderma reesei*) with activity of 70 FPU/mL, supplemented with β-glucosidase (Novozyme 188, produced by *Aspergillus niger*) with activity of 122 CBU/mL. The filter paper unit (FPU) was used to define the enzyme activity that will produce reducing sugar equivalent to 2 mg of glucose [18] and 1 CBU was defined as the amount of enzyme that forms 2 µmol of glucose per min from cellobiose. Enzymatic hydrolysis experiments were carried out in 250 mL bottles. Each bottle was loaded with 1% w/w effective cellulose content, 1% w/v yeast extract, 2% w/v peptone and 0.05 M citrate buffer (pH 4.8) in a final working weight of 50 grams and autoclaved at 121 °C for 15 min. After cooling at room temperature, cellulase enzyme was added to each of the bottles at a dose of 25 FPU/g cellulose and supplemented with β-glucosidase at a dose of 60 CBU/g cellulose to avoid inhibition due to cellobiose accumulation. The reaction mixtures were incubated in a rotary shaker set at 150 rpm and 50 °C for 168 hours. Samples (1 mL) were taken after 0, 3, 24, 48, 72, 96, 120, 144 and 168 hours and stored in capped tubes at –20 °C until used. The capped tubes were boiled in a water bath for exactly 5 min to inactivate cellulase before being chilled on ice. Hydrolyzed samples were centrifuged at 10,000 rpm for 5 min. The supernatants were recovered for reducing sugars analysis at least in triplicate.

### 2.5. Analysis Methods

#### 2.5.1. Analysis of Chemical Composition

The total solids content (also called the percent dry weight) was determined according to the Laboratory Analytical Procedure No.001 (LAP-001) from the National Renewable Energy Laboratory (NREL) [19]. The extractives were removed from analyzed samples by Soxhlet-extraction with ethanol-acetone (1:2 v/v) for 6 h and dried at 103 ± 2 °C for 24 h, according to procedure adapted from TAPPI standard T 204 om-97. The percentage of acid-insoluble lignin was determined according to TAPPI procedure (T224 om-88): a 1 g sample was treated with 72% sulfuric acid (15 mL) and stirred frequently for 2 h at room temperature. After 2 h, the sulfuric acid was diluted with distilled water (560 mL) to obtain the sulfuric acid at a 3% concentration. The solution was then boiled for 4 h, then filtered with distilled water, dried at 103 ± 2 °C for 24 h, and finally weighed. The holocellulose content was determined according to DIN 2403. A mixture of 80 mL distilled water, 1 mL acetic acid (98%), 3 g sodium chlorite, and 2 g of rubberwood sample was heated in a water bath at 70 °C for one hour. The mixture was stirred every 5 min during this time. Acetic acid (1 mL) and sodium chlorite (3 g) were added each hour for the next 3 hours. After 4 hours, the samples were filtered, washed with methanol 3 times, and then dried at 103 ± 2 °C for 24 h and weighed. The α-cellulose content of rubberwood was determined as the residue insoluble in the 17.5% NaOH solution according to TAPPI 203 om-93. Aqueous NaOH solution (25 mL of 17.5%) was added to a flask containing a sample of holocellulose (1 g) and stirred at 20 °C for 40 min, and then 25 mL of distilled water was added. After 5 min, the residue was filtered, and then 40 mL of 10% acetic acid aqueous solution was added to the residue, filtered and washed with 1 L of boiling water. The residue (α-cellulose), was filtrated, dried at 103 ± 2 °C for 48 h and weighed. The percentage hemicellulose was calculated by subtracting the percent α-cellulose from holocellulose. Dry mass loss was calculated as the percentage of total solids loss after pretreatment. Lignin degradation, cellulose loss and hemicellulose loss were defined as the percentage of lignin, cellulose, and hemicellulose reduction.

#### 2.5.2. Determination of Reducing Sugar

Total reducing sugar was determined by the 3, 5-dinitrosalicylic acid (DNS) method using glucose as the standard [20]. The samples were analyzed using a spectrophotometer (Shimadzu, Columbia, MD, USA) at 540 nm. The absorbance readings were then converted into equivalent sugar concentration (mg/mL) using a standard glucose solution curve. Reducing sugar yield was calculated using the following equation:

(1) $$\text{Reducing}\;\text{sugar}\;\text{yield}\;(\%)\;=\;\frac{\text{Reducing}\;\text{sugar}\;\text{produced}\;\times 0.9\;\times 100}{\text{Amount}\;\text{of}\;\text{H}\;\text{Rubberwood}}$$

where: H = Cellulose and hemicelluloses.

#### 2.5.3. X-ray Diffraction Analysis

The wide angle X-ray diffraction of untreated and treated rubberwood was recorded with a Rigaku Geigeflex Diffractometer with Cu and Kα radiation at 30 kV and 30 mA. The diffraction intensity was measured in the range of 2θ = 10–40° at the speed of 2°/min. The crystallinity index (CrI) was calculated using the intensities of crystalline region at 2θ = 22.5° and amorphous region 2θ = 18°, respectively, using the following equation [21]:

(2) $$\text{Crystallinity}\;\text{Index}\;(\text{CrI})\%\;=\;\frac{\text{Icrystalline}-\text{Iamorphous}}{\text{Icrystalline}}$$

where: Icrystalline = Intensity of crystalline region; Iamorphous = Intensity of amorphous region.

#### 2.5.4. Fourier Transform Infrared Spectroscopy (FT-IR) Analysis

The FT-IR spectra of untreated and treated rubberwood were obtained by direct transmittance using the KBr pellet technique. Spectra were recorded with a Perkin Elmer 1650 FT-IR spectrometer (Perkin Elmer, Waltham, MA, USA). The spectra (4000–500 cm^−1^) were measured at a spectral resolution of 4 cm^−1^ and 64 scans per sample.

#### 2.5.5. Statistical Analysis

All experiments (chemical compositions and sugar yield) were carried out in triplicate and the values are an average of the three values obtained within a 95% confidence level. The effects of biological pretreatment on lignin, hemicellulose, and cellulose reduction during biological pretreatments were analyzed using the Statistical Analysis Software (SAS) program [22].

## 3. Results and Discussion

### 3.1. Effect of Fungal Pretreatment on Chemical Composition

**Figure 1:**
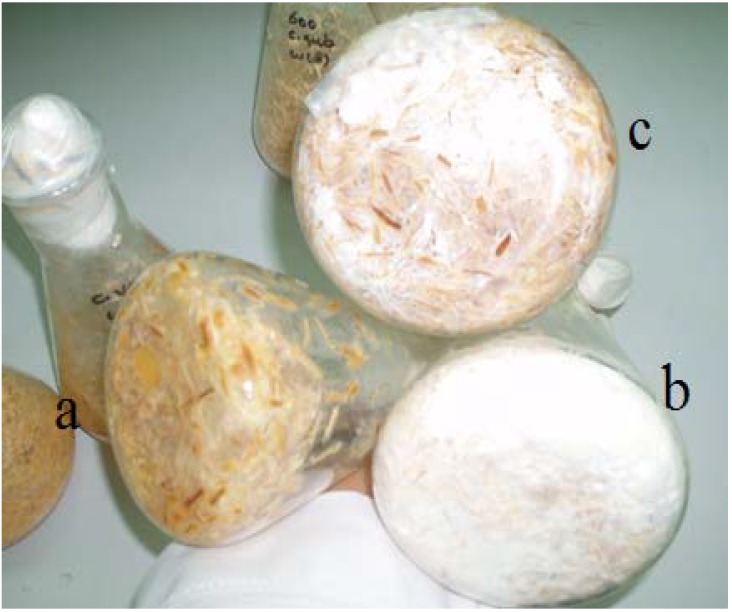
Biological pretreatment of rubberwood after 90 d: (**a**) *C.subvermispora*; (**b**) *T.versicolor*; (**c**) Mixed culture.

Lignin is one of the main components in plant cell walls that limits enzymatic hydrolysis by cross-linking with cellulose and hemicellulose fibers [23]. Reducing the lignin content of the biomass helps to expose the highly ordered crystalline structure of cellulose and facilitates substrate access by hydrolytic enzymes [3]. The effects of biological pretreatment on the rubberwood after cultivation with *C. subvermispora*, *T. versicolor,* and a mixed culture of *C. subvermispora* and *T. versicolor* are shown in Figure 1 and Table 1. All of the components (Table 1) decreased gradually with increasing time. After 90 days cultivation, significant (*p* < 0.05) amounts of lignin were degraded under all fungal pretreatment. The lignin loss of rubberwood caused by *C. subvermispora*, *T. versicolor*, and mixed culture were 45.06%, 34.40%, and 37.68%, respectively. The weight loss of hemicellulose in samples pretreated by *C. subvermispora* (42.08%) and mixed culture (40.83%) was not significantly different (*p* < 0.05 ANOVA, F (3, 6)). Moreover, *T. versicolor* caused the lowest hemicellulose reduction (37.90%) compared to *C. subvermispora* and mixed culture, but both pretreatment by *T. versicolor* and mixed culture caused a significant (*p* < 0.05) weight loss of cellulose, 32.06% and 24.53%, respectively. However, *C. subvermispora* caused only 9.50% weight loss of cellulose.

This study showed that the selective lignin-degrading fungus *C. subvermispora* had a greater selectivity for lignin degradation. A common measure of delignification efficiency is the selectivity of a fungal pretreatment, defined as the ratio of lignin degradation to cellulose reduction [24]. Higher selectivity means better prospects for preferential delignification [25] and low selectivity value means relatively high cellulose loss during the biological pretreatment. As shown in Table 1, *C. subvermispora* demonstrated better selectivity (4.75) than *T. versicolor* (1.07) and mixed culture (1.53) after 90 days of pretreatment. There was significant increase in the selectivity value of rubberwood pretreated with *C. subvermispora* with increasing pretreatment time, which indicated the fungus had great selective lignin-degrading ability. The selectivity value of rubberwood pretreated with *T. versicolor* and mixed culture decreased at advanced stages of pretreatment, even though it increased during the early stages (60 days). These results indicated that *T. versicolor* and mixed culture preferentially degrade lignin only at the early stage of pretreatment (1.16 and 1.62, respectively) and the selectivity turned into a non-selective degradation with increasing pretreatment time; this is similar to the decay of *Pinus radiata* by *Ganoderma austrael* [26].

**Table 1:** Component losses of rubberwood pretreated by white rot fungi for 30, 60, and 90 days^1^.

**Decay time (day)**	**Selectivity value^2^**	**Weight loss (%)**		
		Lignin	Hemicellulose	Cellulose
*C.subvermispora*				
30	3.65^a^ (0.27)	18.80^a^ ( 0.57)	25.13^c^ (0.69)	5.17^c^ (0.46)
60	4.56^a^ (0.36)	37.30^a^ (0.55)	36.02^a^ (1.48)	8.20^c^ (0.42)
90	4.75^a^ (0.31)	45.06^a^ (0.82)	42.08^a^ (1.16)	9.50^c^ (0.48)
*T. versicolor*				
30	1.08^c^ (0.03)	13.34^c^ (0.41)	28.17^a^ (0.54)	12.3^a^ (0.23)
60	1.16^c^ (0.03)	26.88^c^ (0.74)	33.20^b^ (1.30)	23.12^a^ (0.53)
90	1.07^c^ (0.03)	34.40^c^ (0.18)	37.90^b^ (0.58)	32.06^a^ (0.69)
Mixed culture				
30	1.76^b^ (0.15)	15.34^b^ (0.3)	25.52^b^ (0.13)	8.70^b^ (0.12)
60	1.62^b^ (0.02)	28.75^b^ (0.48)	32.36^b^ (0.79)	17.70^b^ (0.45)
90	1.53^b^ (0.03)	37.68^b^ (0.37)	40.83^a^ (0.36)	24.53^b^ (0.40)

^1^ standard deviations of three replicates in parentheses; letters on the right side of the data in the same column indicated significant levels (P < 0.05 ANOVA, F(3,6)); ^2^ selectivity value = Lignin loss/Cellulose loss.

### 3.2. X-ray Diffraction

The minimum intensity of diffraction of the (101) lattice peak at 2θ equal to 18° represents the amorphous cellulose regions and the maximum intensity of the (200) lattice peak at 2θ equal to 22° and 23° represents crystalline cellulose regions. Table 2 presents the data of crystallinity index for untreated and pretreated rubberwood samples with white rot fungi calculated by Segal’s empirical method [21]. As shown in Table 2, all fungi showed increases in the crystallinity index, compared to the untreated wood during pretreatment time. Percent crystallinity index (CrI %) for *C. subvermispora*, *T. versicolor*, and mixed culture had increased after 60 days of decay (65.84%, 61.19%, and 62.38%, respectively). By 90 days, the percentage of the crystallinity of rubberwood decayed by *T. versicolor* and mixed culture had decreased to 51.68% and 52.14%, respectively, relative to wood treated with *C. subvermispora*. The percentage of crystallinity of *C. subvermispora*-treated rubberwood was increased to 66.71% at 90 days. This may be attributed to the greater weight loss of lignin and hemicellulose components after treatment with *T. versicolor* and mixed culture.

In lignocellulolosic material, hemicellulose and lignin are considered to be amorphous components, while cellulose is considered to be the crystalline component [27]. In general, 70% of native cellulose is in the crystalline portion [23]. In the present study, the crystalline portion of the untreated rubberwood was 43.12% and this value increased dramatically after cultivation with fungi. According to Alemdar and Sain [28], the crystallinity index of the pretreated sample was higher because of the absence of hemicellulose and lignin, which are amorphous materials. Analysis of percent crystallinity values in pretreated rubberwood revealed that relative percent crystallinity for wood decayed by *C. subvermispora*, *T. versicolor*, and mixed culture increased by 60 days followed by a decrease as the decay progressed for *T. versicolor* and mixed culture. These findings have been previously observed by researchers examining wood decayed by fungi [29,30] and wood treated chemically to extract hemicellulose [31] and have been attributed to the initial removal of hemicelluloses and other amorphous materials. The high crystallinity of the pretreated sample with *C. subvermispora* indicates that this fungus was able to dramatically remove the lignin and hemicellulose component of rubberwood rather than degrading cellulose. After 60 days, the crystallinity index began to decrease for *T. versicolor* and the mixed culture, possibly due to continuing fungal attack on the crystalline cellulose only after degradation of the more readily available amorphous nutrient sources. However, the crystallinity index for all fungi remained above the level of the controls after 90 days.

**Table 2:** Crystallinity index of untreated and fungal-treated rubberwood after 30, 60, and 90 days.

**Sample**	**CrI (%)**		
	30 d	60 d	90 d
Untreated	43.12	43.12	43.12
*C. subvermispora*	52.37	65.84	66.71
*T. versicolor*	55.48	61.19	51.68
Mixed culture	52.60	62.38	52.14

### 3.3. FT-IR Analysis

#### 3.3.1. Undecayed Rubberwood

**Figure 2:**
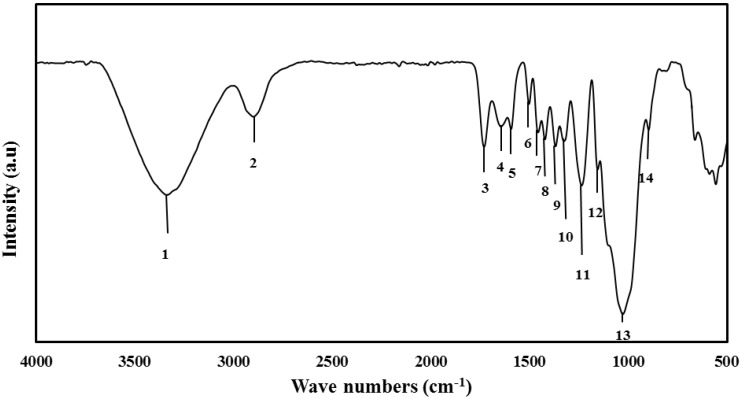
FT-IR spectroscopy of undecayed rubberwood.

FT-IR spectroscopy was used to demonstrate the physical structures and functional groups of the lignocellulosic materials. FT-IR spectroscopy of undecayed rubberwood is shown in Figure 2. The absorbance peaks in the 3400–3300 cm^−1^ (1) region were attributed to the stretching of O-H groups, whereas those around 2900–2800 cm^−1^ (2) were due to the stretching of C-H [32]. The peak located at 1735 cm^−1^ (3) was assigned to the C=O stretching of the acetyl group in hemicellulose [33]. The peaks in the region between 1620 and 1650 cm^−1^ (4 and 5) for all samples were characterized by the absorbed water [34]. The absorbance at 1504 cm^−1^ (6) is attributed to aromatic skeletal vibrations in lignin [33]. The peaks located at 1428 and 1458 cm^−1^ (7 and 8) were assigned to the C-H deformation in lignin and carbohydrates [35]. The peaks observed in the range 1380–1320 cm^−1^ (9 and 10) in all samples were attributed to the bending vibration of C-H and C-O groups of the aromatic ring in polysaccharides [34]. The absorption located at 1234 cm^−1^ (11) is caused by O-H phenolic in lignin [36]. The absorbances at 1158 and 898 (12 and 14) cm^−1^ correspond to C-O-C vibration in cellulose and hemicellulose, and C-H deformation in cellulose, respectively [33]. The C-O-C pyranose ring skeletal vibrations occur in the region 1102–1024 cm^−1^ (13) [37]. The peaks below 898 cm^−1^ are of little importance in the characterization of cellulose.

#### 3.3.2. Comparison of Decayed Rubberwood by *C. Subvermispora*, *T. Versicolor*, and Mixed Culture

**Figure 3:**
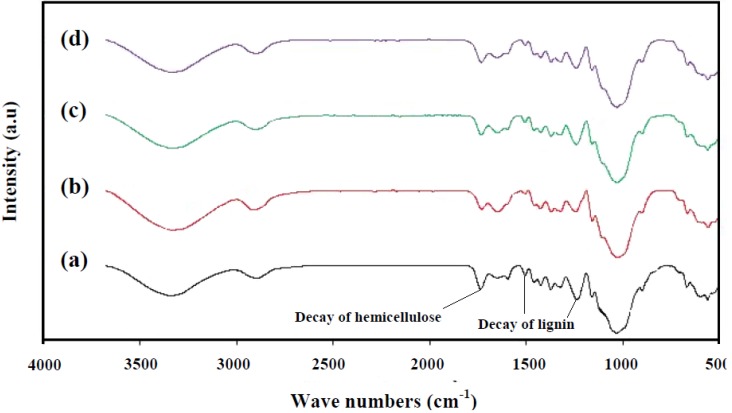
FT-IR spectra of undecayed and decayed rubberwood samples by white rot fungi: (**a**) undecayed wood; **(b**) decayed by mixed culture; (**c**) decayed by *T. versicolo*; (**d**) decayed by *C.subvermispora* for 90 d.

Chemical changes in rubberwood decayed by *C. subvermispora*, *T. versicolor*, and mixed culture were also analyzed using FT-IR spectroscopy (Figure 3). The intensities of carbohydrate bands at 1369, and 897 cm^−1^ were slightly decreased. The constant intensity of the carbohydrate band at 1158 cm^−1^ is remarkable. In particular, the effect of fungal attack on the wood is clearly noticeable by increasing intensity of the 1647 cm^−1^ band (conjugated carbonyl groups, mainly originating from lignin) and the significant decreasing intensities at 1593, 1504, and 1234 cm^−1^ with exposure time [38]. As expected, the intensity of lignin peaks significantly decreased compared with carbohydrate as a result of degradation by white rots, indicating the preferential nature of these white rots. The largest decrease in the intensity of lignin and hemicellulose peaks was observed in case of *C. subvermispora* (Figure 3). From FT-IR spectra analysis, it could be concluded that *C. subvermispora* improved the degradation of lignin but it should have little effect on degradation of carbohydrates which result from a selective lignin removal. These findings are in agreement with Ferraz [26].

### 3.4. Effect of Pretreatment Time on Enzymatic Hydrolysis

**Figure 4:**
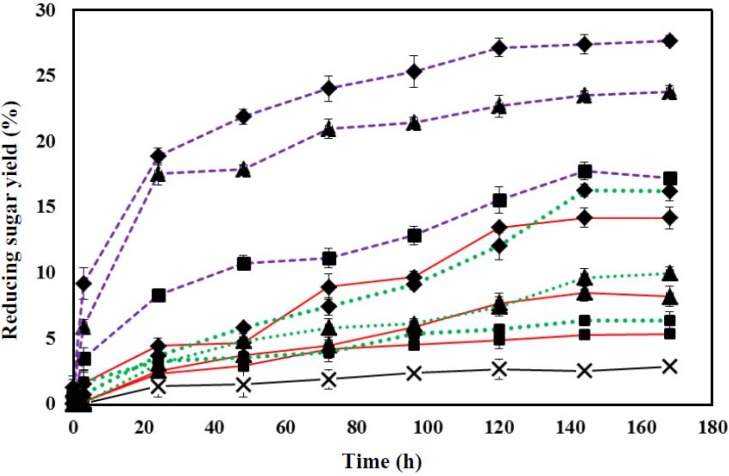
Time course of reducing sugar yield (%) during the hydrolysis of rubberwood (**a)** untreated (×); (**b**) pretreated with *C. subvermispora* (---); (**c**) *T. versicolor* (─); (**d**) mixed culture (∙∙∙) for 30 (■), 60 (▲), and 90 (♦) days. Error bars represent standard error.

To evaluate the effect of pretreatment with *C. subvermispora*, *T. versicolor*, and mixed culture on enzymatic hydrolysis of pretreated rubberwood, we determined fermentable sugar yields after enzymatic hydrolysis for 0, 3, 24, 48, 72, 96, 120, 144, and 168 hours. As expected, rubberwood without pretreatment was much more resistant to enzymatic hydrolysis, producing only 2.88% fermentable sugar yield after 168 hours of hydrolysis (Figure 4). Higher sugar yields were achieved from the rubberwood pretreated with white rot fungi. The reducing sugar yields from samples treated for 30 days with *C. subvermispora*, *T. versicolor*, and mixed culture were 17.23%, 5.34%, and 6.40%, respectively. The reducing sugar yields increased with the cultivation time beyond 30 days, reaching 23.80%, 9.96%, and 8.21% for samples pretreated with *C. subvermispora*, *T. versicolor*, and mixed culture, respectively at 60 days. Further increases were observed when the cultivation time was further extended to 90 days. The highest reducing sugar yields reached about 27.67%, 16.23%, and 14.20% for samples pretreated with *C. subvermispora*, *T. versicolor*, and mixed culture, respectively. These results show that the enzymatic hydrolysis yield of rubberwood is considerably affected by the cultivation time, and the reducing sugar yield heavily depends on the extent of delignification and hemicellulose removal from the lignocellulosic materials due to removal of the physical protective coat of cellulose and, consequently, the improved cellulose digestibility [39]. This explains why samples pretreated by *C. subvermispora* resulted in high sugar yield (27.67%). It was reported that after 120 days of cultivation by a newly isolated fungus, *Echinodontium taxodii* 2538 on two native woods: *Chinese willow* (hard wood) and *China-fair* (soft wood), the enzymatic hydrolysis yield showed significant increases (4.7-fold for hard wood and 3-fold for soft wood) [4]. By contrast, pretreatment of rubberwood (hard wood) by *C. subvermispora*, *T. versicolor*, and mixed culture resulted in much higher enzymatic hydrolysis yields (9.6-fold, 4.9-fold, and 5.6-fold, respectively) during a relatively short degradation period. Lee [40] reported lower sugar yields (21.01%, 14.91%, and 15.03%) from soft wood *Pinus densiflora* pretreated with *Stereum hirsutum*, *Polyporus brumalis*, and *Ceriporia lacerate*, respectively, compared to rubberwood treated with *C. subvermispora* for 72 h at 50 °C in 2007. However, the sugar yield obtained from wood treated with *C. subvermispora* (230.6 mg sugar/g rubberwood) in this study is comparable with that obtained by Zhang [41] (232.2 mg sugar/g bamboo) but in much shortened pretreatment time (90 days compared to 60–120 days by Zhang).

### 3.5. Relationship between Lignin Content and Reducing Sugar Yield

**Figure 5:**
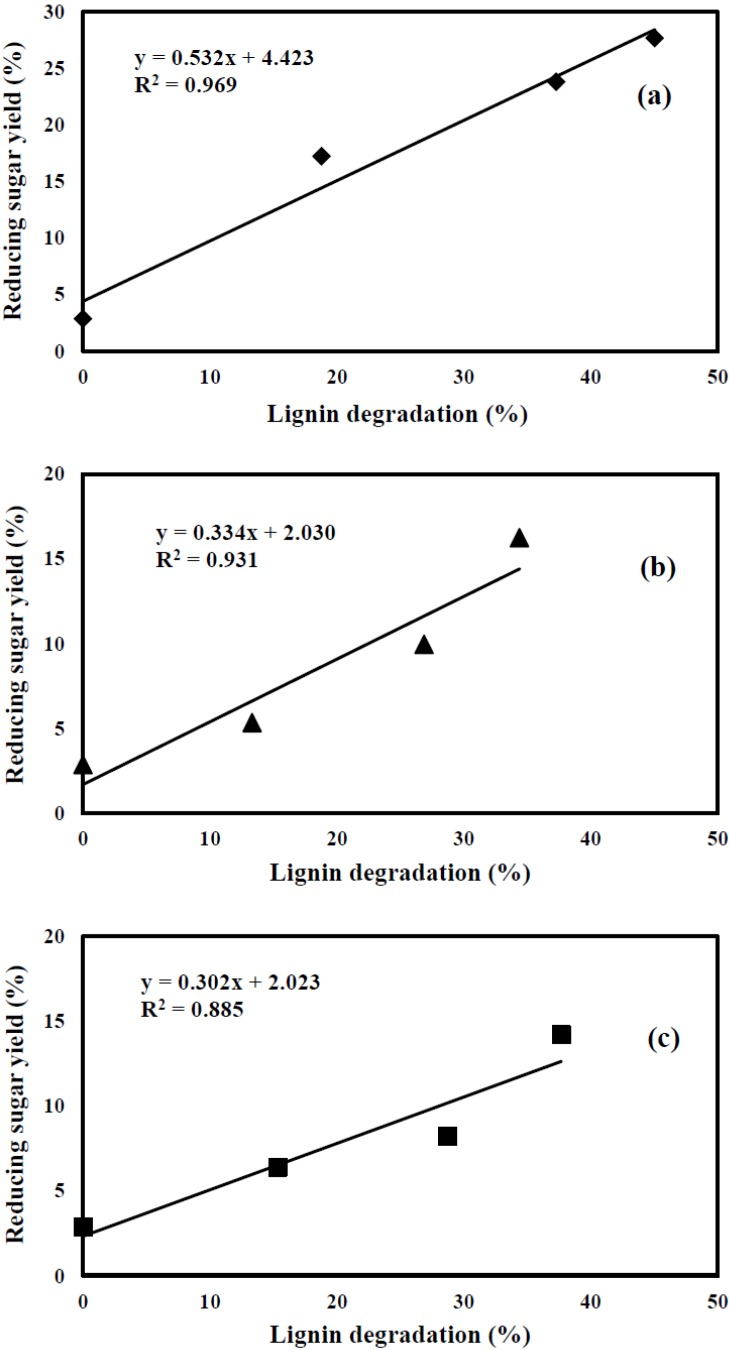
Effect of lignin degradation on enzymatic hydrolysis of rubberwood pretreated with (**a**) *C. subvermispora*; (**b**) *T. versicolor*; (**c**) mixed culture. Reducing sugar yield (%) was obtained after 168 h enzymatic hydrolysis.

It is well recognized in the literature that lignin content and distribution have an impact on the enzymatic hydrolysis [42]. The degradation of lignin after fungal pretreatment can increase pore sizes in the substrate and provide a more accessible surface area to cellulase. Lignin in rubberwood was degraded by pretreatment with *C. subvermispora*, *T. versicolor*, and mixed culture, depending on pretreatment time as shown in Table 1. Figure 5 shows the relationship between lignin content and reducing sugar yield. As it is shown in Figure 5, there is a linear correlation between lignin degradation and reducing sugar yield for rubberwood pretreated with *C. subvermispora* (R^2^ = 0.969). As the lignin degradation increased from 0 (untreated) to 45.06%, the reducing sugar yield increased from 2.88 to 27.67% for the sample treated for 90 days. Moreover, reducing sugar yield of pretreated samples with *T.versicolor* and mixed culture were linearly related to lignin degradation (R^2^ = 0.931 and R^2^ = 0.885, respectively). Compared to mixed culture and *T.versicolor* pretreatment, *C. subvermispora* gave higher regression, which might be attributed to simultaneous holocellulose and lignin degradation during fungal pretreatment. Furthermore, some studies demonstrate the correlation between reducing sugar yield and the lignin content of fungal pretreated biomass [41,43], thus indicating cellulose digestibility can be potentially enhanced by preferential degradation of lignin.

## 4. Conclusions

Solid-state cultivation with *C. subvermispora* was the most preferable solid state pretreatment resulting in 45.06% lignin and 42.08% hemicellulose degradation and minimal cellulose loss (9.50%) compared with *T. versicolor* and combination of both fungi over a period of 90 days. The X-ray analysis exhibited a higher crystallinity for the all fungi-treated samples compared with controls. However, the fungal pretreated sample with *C. subvermispora* showed the highest crystallinity after 90 days. It increased from 43.12% at raw rubberwood, to 66.71% for a pretreated sample after 90 days. The FT-IR results demonstrated that the intensity of lignin peaks significantly decreased compared with carbohydrate as a result of degradation by white rots, thereby indicating the preferential nature of these white rots. The highest decrease in lignin and hemicellulose degradation was observed in the case of *C. subvermispora.* Considering the cellulose content (53%), the potential reducing sugar of rubberwood using biological pretreatment by *C. subvermispora* are 0.230 g of ethanol per g of rubberwood after 90 days.
